# Clinical study of anatomical ACL reconstruction using a rounded rectangular dilator

**DOI:** 10.1186/s12891-020-03913-y

**Published:** 2021-01-07

**Authors:** Junsuke Nakase, Yasushi Takata, Kengo Shimozaki, Kazuki Asai, Rikuto Yoshimizu, Mitsuhiro Kimura, Hiroyuki Tsuchiya

**Affiliations:** Department of Orthopaedic Surgery, Graduate School of Medical Sciences, Kanazawa University, 13-1 Takaramachi, 920-0934 Kanazawa, Japan

**Keywords:** ACL reconstruction, Rounded rectangular bone tunnel, Anatomical reconstruction, Single‐bundle reconstruction

## Abstract

**Background:**

The aim of this study was that to compare clinical results between the rounded rectangular femoral tunnel ACL reconstruction (RFTR) and the conventional round femoral tunnel ACL reconstruction using a hamstring tendon. The hypothesis was that ACL reconstruction performed using the rounded rectangular dilator technique was better than that performed using the conventional round femoral tunnel technique in terms of clinical results and bone tunnel enlargement.

**Methods:**

We conducted retrospective study. After exclusions, 40 patients were included in the conventional anatomical single-bundle ACL reconstruction (ASBR) group and 40 patients were included in the RFTR group. The evaluation items were knee stability, Lysholm knee score, IKDC subjective score at 2 years after surgery and bone tunnel enlargement.

**Results:**

The RFTR group had a larger femoral tunnel area (average area, 53.1 ± 4.0 mm^2^ vs. 46.1 ± 7.0 mm^2^; *P* < 0.01), better anteroposterior stability, and higher Lysholm scores than the ASBR group (average side-to-side difference for anterior tibial translation, 0.6 ± 0.8 mm vs. 1.6 ± 1.4 mm; *P* < 0.01; average Lysholm score, 98.5 ± 2.1 vs. 97.5 ± 3.5; *P* < 0.01). Further, bone tunnel enlargement ratio was significantly lower in the RFTR group (73 ± 38% vs. 107 ± 41%; *P* < 0.01).

**Conclusions:**

We designed and developed an original rounded rectangular dilator to perform a novel ACL surgery. This technique can create a larger bone tunnel and improve clinical results than the conventional round anatomical single-bundle ACL reconstruction.

## Background

**Fig. 1:**
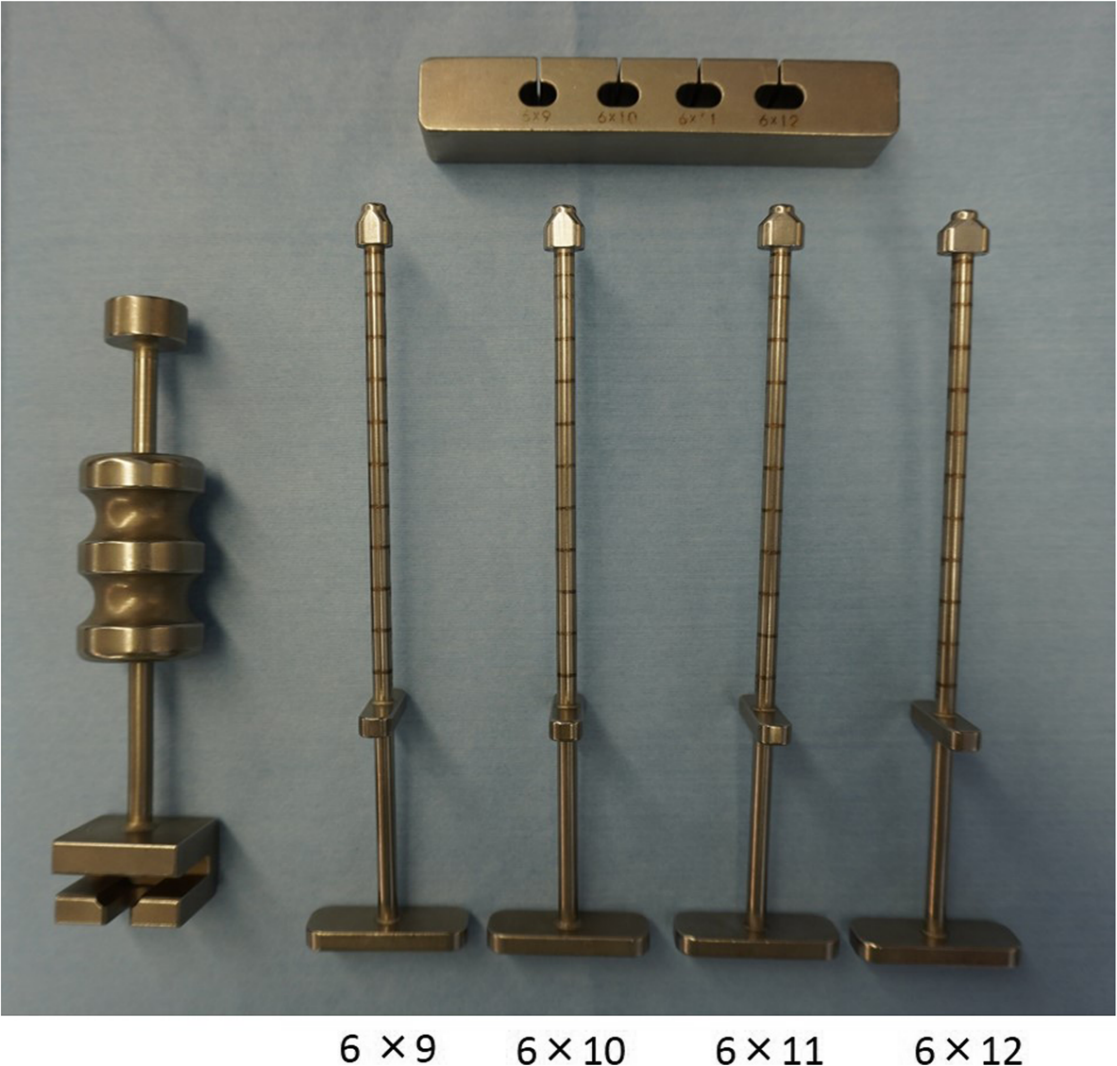
Rounded rectangle dilator and sizing book set. This set includes 4 types from 6 × 9 mm to 6 × 12 mm

In anterior cruciate ligament (ACL) reconstruction, there is consensus regarding the creation of femoral and tibial tunnels within the anatomical insertion area of the ACL to obtain good clinical results [1]. There have been various opinions on the ACL’s anatomy since the report published by Girgis et al. in 1975 [2–5]. Recently, Smigielski et al. reported that the ACL, including its femoral and tibial insertions, appears to be flat and “ribbon-like” after the removal of the synovial membrane [6]. After this report, instead of double-bundle ACL reconstruction and the conventional single-bundle ACL reconstruction, new anatomical single-bundle techniques using a hamstring tendon (oval femoral tunnel [7] and rectangular femoral tunnel [8]) have been developed and reported. Observing the cross-sectional shape of the quadrupled hamstring harvested at surgery, we felt that it approximated an oval shape. To prove this, we performed cross-sectional measurements of quadrupled hamstring from fresh cadaveric knees. We realized that the cross-sectional shape of the femoral insertion of the quadrupled hamstring graft, which is used for anatomical single-bundle ACL reconstruction, is oval, not round [9]. It was revealed that single-bundle reconstruction using a thin graft with a diameter of less than 8 mm increases the incidence of repeat surgery [10, 11]. Therefore, while the surgeon may have intended to create a large bone tunnel, the region’s anatomical characteristics mean that anatomical single-bundle ACL reconstruction using a round drill cannot increase the size of the femoral tunnel without roof impingement or breakage of the posterior wall of the femoral condyle. The anatomy of the ACL attachment, the cross-sectional shape of the graft, and the mechanical characteristics of the tendon led us to the idea of creating a rounded rectangular femoral tunnel. Thus, we designed and developed an original rounded rectangle tendon diameter tester and a dilator for the new anatomical single-bundle ACL reconstruction in 2012 (Fig. 1). In 2016, we reported that this technique – “rounded rectangular femoral tunnel ACLR” (RFTR) – did not involve serious intraoperative complications and was easy and reproducible [12]. The concept of this technique is to create a femoral tunnel parallel to the lateral intercondylar ridge and reconstruct the tendon–bone junction in a straight manner to mimic the normal anatomy. In addition to the safety and accuracy of this technique, we also investigated the gap between the tendon and the bone tunnel using a fresh cadaveric study and demonstrated that this gap was smaller than that of the conventional round bone tunnel [13]. Based on this, we realized that this technique can accommodate a large graft without roof impingement with good clinical results. We report the clinical results of this technique 2 years after ACL reconstruction.

This study was conducted to compare clinical results between the RFTR and the conventional round femoral tunnel technique in ACL reconstruction using a hamstring tendon. The hypothesis was that ACL reconstruction performed using the rounded rectangular dilator technique was better than that performed using the conventional round femoral tunnel technique in terms of clinical results and bone tunnel enlargement.

## Methods

In this retrospective study, written informed consent was obtained from all patients included. This study design was reviewed and approved by Kanazawa University Medical Ethics Review Committee (approval No. 1842).

The subjects included in the study were selected from a group of patients who underwent ACL reconstruction in our hospital between April 2011 and March 2016. Diagnosis of ACL injury was made depending on a history of knee injury and the results of the Lachman and pivot shift tests, as well as a side-to-side difference of more than 3 mm measured using the KT-1000 arthrometer (MeDmetric, San Diego, CA). All patients underwent magnetic resonance imaging to confirm the diagnosis of ACL tear. All procedures were conducted by a single surgeon. The inclusion criteria were as follows: individuals who were aged between 13 and 60 years, who had an acute isolated ACL tear within 6 months after injury, with no history of knee surgery, and who underwent single-bundle ACL reconstruction with a hamstring tendon autograft. The exclusion criteria were as follows: patients who had an Outerbridge classification [14] of cartilage injury of greater severity than grade II and underwent meniscal repair with the inside-out technique.

Forty-eight patients admitted between April 2011 and March 2013 were assigned to the conventional anatomical single-bundle ACL reconstruction (ASBR) group and 50 patients admitted between June 2013 and March 2016, whose age and sex matched those of the ASBR group, were categorized as the RFTR group. Eighteen patients were excluded from this study. In the ASBR group, four patients underwent meniscal repair using the inside-out technique. In the RFTR group, one patient had grade III cartilage injury and six patients underwent meniscal repair using the inside-out technique. Four patients in the ASBR group and 3 patients in the RFTR group were lost to follow-up. Therefore, of the remaining 80 patients, 40 were included in the ASBR group and 40 were included in the RFTR group. No significant differences were detected between the two groups with regard to demographic characteristics (Table 1).

**Table 1:** Patients’ demographic data

	RFTR (40)	ASBR (40)	*P* value
Age (years)	24.8 ± 11.1	25.3 ± 10.0	0.806
Sex	M 15, F 25	M 15, F 25	1
Height(cm)	163.8 ± 8.8	162.2 ± 7.2	0.806
Body weight(kg)	60.8 ± 11.9	58.4 ± 9.3	0.296
Injury-surgery interval (weeks)	12.0 ± 6.6	11.0 ± 6.5	0.521
Medial meniscus tear	6	7	
Partial meniscectomy	0	1	
All inside meniscal repair	6	6	
Lateral meniscus tear	10	11	
Partial meniscectomy	3	5	
All inside meniscal repair	7	6	

Values were expressed as mean ±standard deviation

### Surgical technique

We performed ACL reconstruction under general anesthesia with nerve block. The semitendinosus tendon was harvested using an open tendon stripper. When the fourfold semitendinosus graft size was less than 6 × 9 mm or 7 mm, we harvested the gracilis tendon.

### Femoral tunnel

**Fig. 2:**
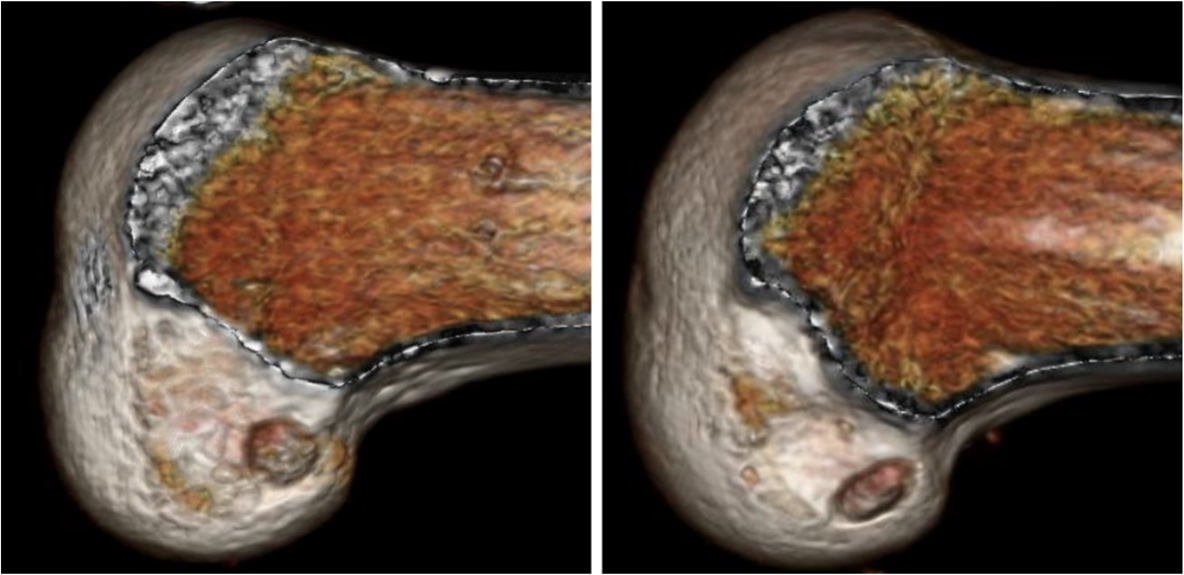
Postoperative 3DCT image. Left: Conventional anatomical single-bundle ACL reconstruction. Femoral bone tunnel size is 8 mm. Right: Rounded rectangular femoral tunnel ACL reconstruction. Femoral bone tunnel size is 6 × 10 mm

The femoral tunnel was created before the tibial tunnel through an additional low anteromedial portal. Using the lateral intercondylar ridge as an anatomical landmark, a mark was made at the center of the femoral insertion. With the knee in full flexion, a RetroButton Drill Pin (Arthrex, Naples, FL) was inserted through the low anteromedial portal, penetrating the lateral side of the thigh, to create a femoral tunnel of 3.5-mm diameter, and a reamer (7–9 mm) was used to create the conventional round tunnel at 15-mm depth (Fig. 2). To create the rounded rectangular femoral tunnel, we drilled to a length of 15 mm using a 6-mm drill tip through a RetroButton Drill Pin. Subsequently, we used the original rounded rectangle dilators, which are available in various sizes. In all cases, we dilated the entire 15 mm of the femoral tunnel (Fig. 2).

### Tibial tunnel

The tibial tunnel was drilled with a tibial guide set at a 50° angle, and the tip of the aimer was positioned to be 3–4 mm anterior to the posterior border of the anterior horn of the lateral meniscus, and directly anteromedial to the center of the tibial attachment of the ACL. The tunnel was then drilled according to the diameter of the graft with a conventional drill bit in both groups.

### Graft fixation

The graft was inserted through the tibial tunnel and was looped over a TightRope (Arthrex) for femoral fixation. After the button was flipped, the graft was manually pulled backwards and the joint was moved several times through the full range of motion. The other end of the graft was fixed using a Double Spike Plate (Smith and Nephew, Andover, MA) and a screw, and the initial graft tension was set to 40 N at 20° of knee flexion in both groups.

### Postoperative rehabilitation

Full weight bearing and walking, depending on pain, were allowed from the day after the surgery. A physical therapist performed range of motion training on the day after the surgery. The subjects wore an extension brace for 1 week after the surgery and attached the soft brace for the subsequent 4 months. The subjects walked using crutches for 1 month, started jogging 3 months after the surgery, started running 5 months after the surgery, and returned to sports activities at least 6 months after the surgery.

### Clinical evaluations

**Fig. 3:**
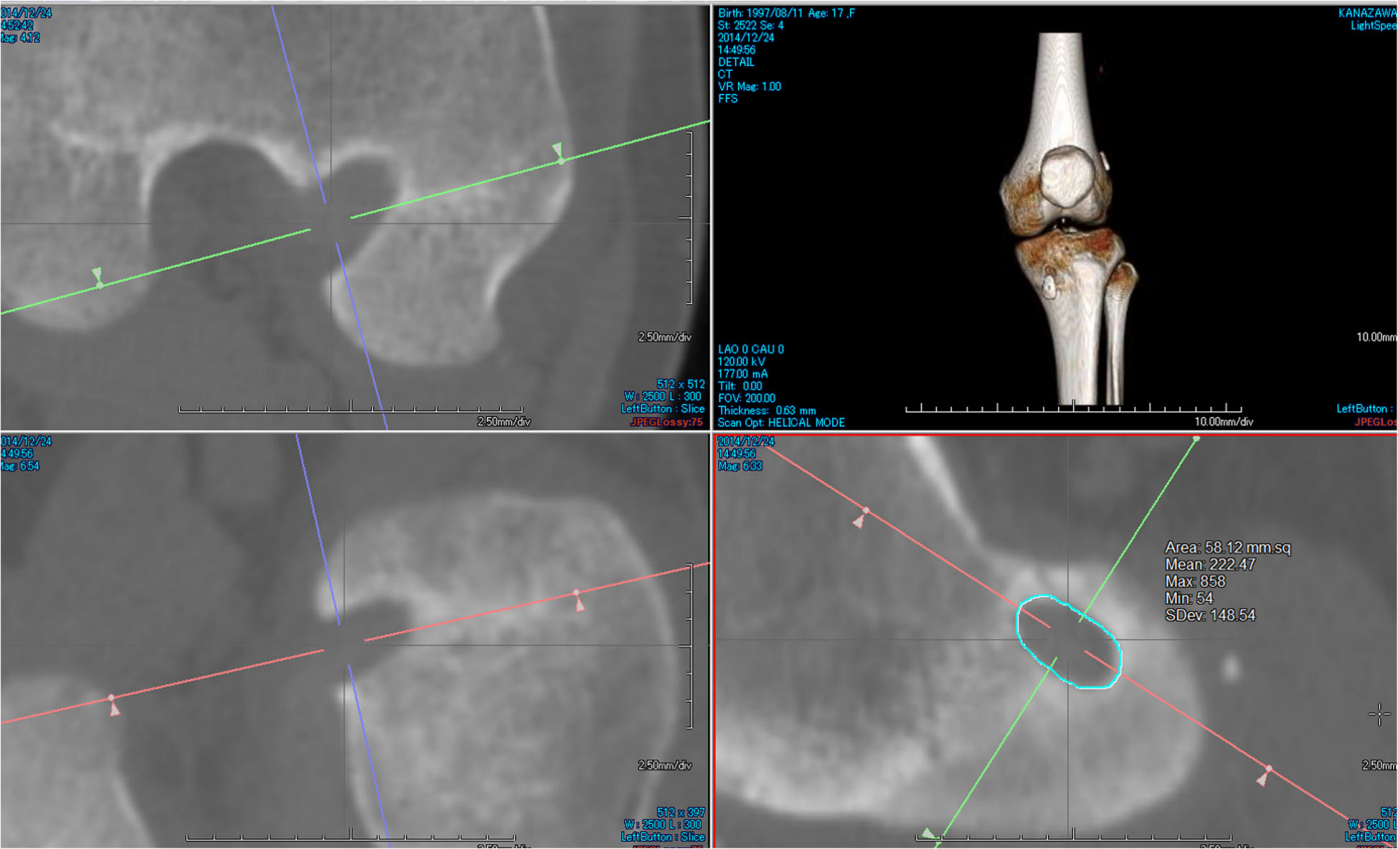
Representative case. Bone tunnel area was calculated using a 2-mm-deep slice parallel to the femoral bone tunnel opening within the intercondylar wall of the lateral condyle. In this case, 1 week postoperative CT scan of the RFTR group, the bone tunnel area was 58.1 mm^2^

An independent orthopedic surgeon performed all clinical evaluations at 2 years after the surgery. For the evaluation of knee stability, the anterior laxity of the normal and reconstructed knees was measured with the KT-1000 arthrometer. Differences between both knees were calculated. At least three measurements were performed on each knee, and the mean value was recorded. Passive knee range of motion was measured with a goniometer and compared with that of the contralateral knee. The pivot-shift test was performed manually and graded as 0 (negative), 1 (glide), 2 (clunk), and 3 (gross). All subjects were asked to complete self-reported knee function surveys, including the Lysholm knee score [15] and the International Knee Documentation Committee (IKDC) questionnaire [16]. To calculate bone tunnel enlargement, we compared computed tomography (CT) images taken at 1 week after the surgery with those taken at 3 months after the surgery. We identified the slice of the femoral opening using the AquarisNET (TeraRecon Inc., Foster City, CA) program, made a cross-sectional slice of 2-mm depth near the opening of the bone tunnels, and measured the femoral bone tunnel area. We traced the tunnel wall within the sclerotic bony margin and measured the tunnel area that was surrounded by trace lines (Fig. 3). Measured tunnel areas were compared within each period, and the bone tunnel enlargement ratio was calculated according to the following equation: bone tunnel enlargement ration (%) = (tunnel area 3 months after surgery – tunnel area 1 week after surgery) / tunnel area 1 week after surgery × 100.

### Statistical analysis

All data were analyzed using SPSS 23.0 (IBM SPSS Statistics 23). For continuous and normally distributed data, Student’s t-test was applied to determine significant differences between both groups. The Pearson chi-square test was performed to compare dichotomous variables. The significance level was set at 0.05 (two-sided) with a 95% confidence interval. Sample size was calculated using G-Power 3.1 (effect size 0.8, α-error 0.05, and target power 0.95) and a minimum of 35 subjects per group was recommended.

## Results

The RFTR group had a larger femoral tunnel area than the ASBR group (average area, 53.1 ± 4.0 mm^2^ vs. 46.1 ± 7.0 mm^2^; *P* < 0.01). The RFTR group had better anteroposterior stability and higher Lysholm scores than the ASBR group (average side-to-side difference for anterior tibial translation, 0.6 ± 0.8 mm vs. 1.6 ± 1.4 mm; *P* < 0.01; average Lysholm score, 98.5 ± 2.1 vs. 97.5 ± 3.5; *P* < 0.01). Differences in rotational laxity between the groups were significant (negative pivot shift, 94.3% vs. 92.3%; *P* < 0.01). No significant difference was noted in the IKDC subjective score between the two groups. The RFTR group had significantly larger femoral bone tunnels immediately after surgery; however, no significant differences were observed in bone tunnel area on the 3-month postoperative CT (106.2 ± 23.8 mm^2^ vs. 102.1 ± 24.8 mm^2^; *p* = 0.264). The bone tunnel enlargement ratio was significantly lower in the RFTR group (73 ± 38% vs. 107 ± 41%; *P* < 0.01) (Table 2). No surgical site infections were detected in both groups. Two patients experienced partial posterior tunnel wall blowout; we were able to fix the graft using the standard technique. No other intraoperative/postoperative complications were observed.

**Table 2:** Comparison of the clinical results

	RFTR (40)	ASBR (40)	*P* value
Graft (STG, ST)	16, 24	16, 24	1
Femoral tunnel size (mm)	6 × 9: 5	7: 16	
	6 × 10: 25	8: 22	
	6 × 11: 9	9: 2	
	6 × 12: 1		
Femoral tunnel area (mm^2^)	53.1 ± 4.0	46.1 ± 7.0	< 0.01
Anteroposterior knee laxity (mm)	0.6 ± 0.8	1.6 ± 1.4	< 0.01
Negative pivot shift (%)	92.3	82.5	< 0.01
Lysholm score	98.5 ± 2.1	97.5 ± 3.5	< 0.01
IKDC subjective score	94.3 ± 6.7	92.3 ± 11.2	0.293
Femoral tunnel area 1 week after surgery (mm^2^)	69.8 ± 18.3	55.3 ± 18.6	< 0.01
Femoral tunnel area 3 months after surgery (mm^2^)	106.2 ± 23.8	102.1 ± 24.8	0.026
Bone tunnel enlargement ratio (%)	73 ± 38%	107 ± 41	< 0.01

Values were expressed as mean ± standard deviation and evaluated using Student’s t test

*ST* Semitendinosis, *G* Gracilis

## Discussion

The most important finding of this study is that we successfully designed and developed an original rounded rectangular dilator to perform a novel anatomical single-bundle ACL reconstruction. This technique created a larger bone tunnel and achieved clinical results superior to those obtained using the conventional round anatomical single-bundle ACL reconstruction.

In recent years, both single-bundle and double-bundle techniques have been commonly used in ACL reconstruction. Several studies have reported that the double-bundle technique of ACL reconstruction could restore knee stability and kinematics to levels closer to those of the native knee than the conventional round single-bundle technique [17–19]. However, there remain concerns regarding the double-bundle technique. One concern is the need to drill four independent tunnels, which doubles the risk of incorrect tunnel placement, and several authors have reported significant tunnel widening after double-bundle ACL reconstruction [20]. In addition, tunnel coalition occurs in double-bundle ACL reconstruction (femoral tunnel 5.1–19%, tibial tunnel 27-56.4% [21–23]), and this influences knee stability and clinical results [24]. Furthermore, double-bundle ACL reconstruction requires longer operative time and creates more extensive bone loss, thereby potentially increasing the difficulty of revision surgery. Thus, attention has been turned toward single-bundle reconstruction with grafts that are placed at the center of the anatomical footprint. Biomechanical studies have shown that double-bundle reconstruction might not have significant advantages over anatomical single-bundle reconstruction [25]. Additionally, the clinical results of a previous study and a meta-analysis demonstrate no significance difference between anatomical double-bundle and single-bundle ACL reconstructions at the 5-year mid-term follow-up [26, 27]. However, in this study, the clinical outcomes of single-bundle ACL reconstruction using a round bone tunnel tended to be poor when the graft was 7 mm. This result is supported by several studies in the literature [10, 11]. Currently, the diameter of the graft is not less than 8 mm, we used 6 × 10 mm as the minimum size, and if smaller, we added the gracilis tendon and reconstructed it [28] (Table 3). Our technique not only enables anatomical reconstruction with a thick tendon graft but also creates a femoral bone tunnel using a dilator, which increases the CT value of the tunnel walls and reduces bone tunnel enlargement [29].

**Table 3:** Area of the round and rounded rectangular bone tunnel

Round (mm)	Area (mm^2^)	Rounded rectangle (mm)	Area (mm^2^)
7	38.4	5 × 9	39.6
7.5	44.2	6 × 9	46.2
8	50.2	6 × 10	52.2
8.5	56.7	6 × 11	58.2
9	63.5	6 × 12	64.2

In this technique, the tibial tunnel forms a round shape. Based on anatomical knowledge, it may be ideal to make the tibial tunnel into a rounded rectangle or C-shape [7, 30]. The shape of the tibial bone tunnel has to be changed if the lateral anterior meniscus is damaged, and this would lead to poor clinical results due to the round bone tunnel. In our clinical study, partial lateral meniscus anterior root injury during this technique occurred in 18% of patients and no patient experienced lateral meniscal extrusion. It is important to keep the surgery as simple and reproducible as possible. We plan to continue mid-term and long-term follow-up.

Wen et al. reported four theories to explain the advantage of the oval femoral tunnel technique [8]. First, an oval femoral tunnel provides a larger surface area for better blood supply from the adjacent cancellous bone surrounding the femoral tunnel. Second, an oval femoral tunnel closely resembles ACL anatomic insertions and restores natural ACL morphology. Third, the grafts used in an oval femoral tunnel should not easily rotate, and this is advantageous for tendon bone healing. Fourth, using the dilator technique, which ensures maximum preservation of the cancellous bone, leads to better mechanical stability [8]. Zhao et al. reported that a flattened bone tunnel accelerated tendon bone healing in the early period after ACL reconstruction in a rabbit model [31]. These theories apply to our technique.

This study has two important limitations. One of the limitations of this study is that it was retrospective study and the surgical technique used differed according to the study period; therefore, the study was not randomized and blinded. However, all clinical evaluations were performed by an independent orthopedic surgeon, and evaluation of the CT scans was performed by an orthopedic surgeon who was blinded to the clinical outcomes. In the future, prospective randomized controlled trials are needed. Second, the postoperative follow-up period was only 2 years and did not account for short- and long-term follow-up. Further research is needed to determine such as osteoarthritis and other possible complications.

## Conclusions

We designed and developed an original rounded rectangular dilator to perform a novel ACL operation. This technique can create a larger bone tunnel and achieve superior clinical results than the conventional round anatomical single-bundle ACL reconstruction.

## Data Availability

The datasets used and analyzed during the current study are available from the corresponding author on reasonable request.

## Abbreviations

ACL: Anterior cruciate ligament
RFTR: Rounded rectangular femoral tunnel ACL reconstruction
ASBR: Conventional anatomical single-bundle ACL reconstruction
IKDC: International Knee Documentation Committee
CT: Computed tomography

## References

[1] Schillhammer CK, Reid JB, Rister J, Jani SS, Marvil SC, Chen AW (2016). Arthroscopy up to date: anterior cruciate ligament anatomy. Arthroscopy.

[2] Girgis FG, Marshall JL, Monajem A (1975). The cruciate ligaments of the knee joint. Anatomical, functional and experimental analysis. Clin Orthop Relat Res.

[3] Hara K, Mochizuki T, Sekiya I, Yamaguchi K, Akita K, Muneta T (2009). Anatomy of normal human anterior cruciate ligament attachments evaluated by divided small bundles. Am J Sports Med.

[4] Mochizuki T, Muneta T, Nagase T, Shirasawa S, Akita KI, Sekiya I (2006). Cadaveric knee observation study for describing anatomic femoral tunnel placement for two-bundle anterior cruciate ligament reconstruction. Arthroscopy.

[5] Iwahashi T, Shino K, Nakata K, Otsubo H, Suzuki T, Amano H (2010). Direct anterior cruciate ligament insertion to the femur assessed by histology and 3-dimensional volume-rendered computed tomography. Arthroscopy.

[6] Śmigielski R, Zdanowicz U, Drwięga M, Ciszek B, Williams A (2016). The anatomy of the anterior cruciate ligament and its relevance to the technique of reconstruction. Bone Joint J.

[7] Fink C, Smigielski R, Siebold R, Abermann E, Herbort M (2020). Anterior cruciate ligament reconstruction using a ribbon-like graft with a c-shaped tibial bone tunnel. Arthrosc Tech.

[8] Wen Z, Zhang H, Yan W, Mohamed SI, Zhao P, Huang X, et al. Oval femoral tunnel technique is superior to the conventional round femoral tunnel technique using the hamstring tendon in anatomical anterior cruciate ligament reconstruction. Knee Surg Sports Traumatol Arthrosc 2019;27. 10.1007/s00167-019-05809-4. [Epub ahead of print]. 10.1007/s00167-019-05809-4 31776627

[9] Oshima T, Nakase J, Numata H, Takata Y, Tsuchiya H (2016). The cross-sectional shape of the fourfold semitendinosus tendon is oval, not round. J Exp Orthop.

[10] Snaebjörnsson T, Senorski EH, Ayeni OR, Alentorn-Geli E, Krupic F, Norberg F (2017). Graft diameter as a predictor for revision anterior cruciate ligament reconstruction and KOOS and EQ-5D values: a cohort study from the Swedish National Knee Ligament Register based on 2240 patients. Am J Sports Med.

[11] Kaeding CC, Pedroza AD, Reinke EK, Huston LJ, Spindler KP, MOON Consortium (2015). Risk factors and predictors of subsequent ACL injury in either knee after ACL reconstruction: prospective analysis of 2488 primary ACL reconstructions from the MOON cohort. Am J Sports Med.

[12] Nakase J, Toratani T, Kosaka M, Ohashi Y, Numata H, Oshima T (2016). Technique of anatomical single bundle ACL reconstruction with rounded rectangle femoral dilator. Knee.

[13] Takata Y, Nakase J, Oshima T, Shimozaki K, Asai K, Tsuchiya H (2018). No difference in the graft shift between a round and a rounded rectangular femoral tunnel for anterior cruciate ligament reconstruction: an experimental study. Arch Orthop Trauma Surg.

[14] Slattery C, Kweon CY (2018). Classifications in brief: outerbridge classification of chondral lesions. Clin Orthop Relat Res.

[15] Tegner Y, Lysholm J (1985). Rating systems in the evaluation of knee ligament injuries. Clin Orthop Relat Res.

[16] van Meer BL, Meuffels DE, Vissers MM, Bierma-Zeinstra SM, Verhaar JA, Terwee CB (2013). Knee Injury and Osteoarthritis Outcome Score or International Knee Documentation Committee Subjective Knee Form: which questionnaire is most useful to monitor patients with an anterior cruciate ligament rupture in the short. term? Arthroscopy.

[17] Komzák M, Hart R, Feranec M, Šmíd P, Kocová R (2018). In vivo knee rotational stability 2 years after double-bundle and anatomic single-bundle ACL reconstruction. Eur J Trauma Emerg Surg.

[18] Tsai AG, Wijdicks CA, Walsh MP, Laprade RF (2010). Comparative kinematic evaluation of all-inside single-bundle and double-bundle anterior cruciate ligament reconstruction: a biomechanical study. Am J Sports Med.

[19] Aglietti P, Giron F, Losco M, Cuomo P, Ciardullo A, Mondanelli N (2010). Comparison between single-and double-bundle anterior cruciate ligament reconstruction: a prospective, randomized, single-blinded clinical trial. Am J Sports Med.

[20] Siebold R (2007). Observations on bone tunnel enlargement after double-bundle anterior cruciate ligament reconstruction. Arthroscopy.

[21] Siebold R, Cafaltzis K (2010). Differentiation between intraoperative and postoperative bone tunnel widening and communication in double-bundle anterior cruciate ligament reconstruction: a prospective study. Arthroscopy.

[22] Kiekara T, Järvelä T, Huhtala H, Moisala AS, Suomalainen P, Paakkala A (2014). Tunnel communication and increased graft signal intensity on magnetic resonance imaging of double-bundle anterior cruciate ligament reconstruction. Arthroscopy.

[23] Kawaguchi Y, Kondo E, Onodera J, Kitamura N, Sasaki T, Yagi T (2013). Tunnel enlargement and coalition after anatomic double-bundle anterior cruciate ligament reconstruction with hamstring tendon autografts: a computed tomography study. Orthop J Sports Med.

[24] Masuda T, Kondo E, Onodera J, Kitamura N, Inoue M, Nakamura E (2018). Effects of remnant tissue preservation on tunnel enlargement after anatomic double-bundle anterior cruciate ligament reconstruction using the hamstring tendon. Orthop J Sports Med.

[25] Kondo E, Merican AM, Yasuda K, Amis AA (2011). Biomechanical comparison of anatomic double-bundle, anatomic single-bundle, and nonanatomic single-bundle anterior cruciate ligament reconstructions. Am J Sports Med.

[26] Chen H, Chen B, Tie K, Fu Z, Chen L (2018). Single-bundle versus double-bundle autologous anterior cruciate ligament reconstruction: a meta-analysis of randomized controlled trials at 5-year minimum follow-up. J Orthop Surg Res.

[27] Karikis I, Desai N, Sernert N, Rostgard-Christensen L, Kartus J (2016). Comparison of anatomic double- and single-bundle techniques for anterior cruciate ligament reconstruction using hamstring tendon autografts: a prospective randomized study with 5-year clinical and radiographic follow-up. Am J Sports Med.

[28] Duerr RA, Garvey KD, Ackermann J, Matzkin EG (2019). Influence of graft diameter on patient reported outcomes after hamstring autograft anterior cruciate ligament reconstruction. Orthop Rev (Pavia).

[29] Takata Y, Nakase J, Numata H, Oshima T, Tsuchiya H (2016). Computed tomography value and tunnel enlargement of round and rounded rectangular femoral bone tunnel for anterior cruciate ligament reconstruction. Arch Orthop Trauma Surg.

[30] Zhang J, Hu X, Liu Z, Zhao F, Ma Y, Ao Y (2019). Anatomical single bundle anterior cruciate ligament reconstruction with rounded rectangle tibial tunnel and oval femoral tunnel: a prospective comparative study versus conventional surgery. Am J Transl Res.

[31] Zhao F, Hu X, Zhang J, Shi W, Ren B, Huang H (2019). A more flattened bone tunnel has a positive effect on tendon-bone healing in the early period after ACL reconstruction. Knee Surg Sports Traumatol Arthrosc.

